# Production and Identification of Wheat-*Agropyron cristatum* 2P Translocation Lines

**DOI:** 10.1371/journal.pone.0145928

**Published:** 2016-01-05

**Authors:** Huanhuan Li, Mingjie Lv, Liqiang Song, Jinpeng Zhang, Ainong Gao, Lihui Li, Weihua Liu

**Affiliations:** National Key Facility for Crop Gene Resources and Genetic Improvement/Institute of Crop Sciences, Chinese Academy of Agricultural Sciences, Beijing, 100081, China; Nanjing Agricultural University, CHINA

## Abstract

*Agropyron cristatum* (L.) Gaertn. (2*n* = 28, **PPPP**), a wild relative of common wheat, possesses many potentially valuable traits that can be transferred to common wheat through breeding programs. The wheat-*A*. *cristatum* disomic addition and translocation lines can be used as bridge materials to introduce alien chromosomal segments to wheat. Wheat-*A*. *cristatum* 2**P** disomic addition line II-9-3 was highly resistant to powdery mildew and leaf rust, which was reported in our previous study. However, some translocation lines induced from II-9-3 have not been reported. In this study, some translocation lines were induced from II-9-3 by ^60^Co-γ irradiation and gametocidal chromosome 2**C** and then identified by cytological methods. Forty-nine wheat-*A*. *cristatum* translocation lines were obtained and various translcoation types were identified by GISH (genomic *in situ* hybridization), such as whole-arm, segmental and intercalary translocations. Dual-color FISH (fluorescent *in situ* hybridization) was applied to identify the wheat chromosomes involved in the translocations, and the results showed that *A*. *cristatum* 2**P** chromosome segments were translocated to the different wheat chromosomes, including 1**A**, 2**A**, 3**A**, 4**A**, 5**A**, 6**A**, 7**A**, 3**B**, 5**B**, 7**B**, 1**D**, 4**D** and 6**D**. Many different types of wheat-*A*. *cristatum* alien translocation lines would be valuable for not only identifying and cloning *A*. *cristatum* 2**P**-related genes and understanding the genetics and breeding effects of the translocation between *A*. *cristatum* chromosome 2**P** and wheat chromosomes, but also providing new germplasm resources for the wheat genetic improvement.

## Introduction

Wheat (*Triticum aestivum* L.), widely planted in different parts of the world, is the third most important cereal behind maize and rice. However, modern breeding was challenged by the narrow genetic variation [1,2], which affected further improvements in wheat yield and quality. Wild relatives of wheat possessed many desirable and valuable traits that could be used as gene resources for wheat improvement [3–5]. For example, *Agropyron cristatum* chromosome 6**P** with the genes controlling large numbers of florets and kernels per spike and multiple fertile tiller numbers per plant [6,7]; *Dasypyrum villosum* chromosome 1**V** including the seed storage protein genes [8]; *Thinopyrum ponticum* containing stem rust resistance gene *Sr43* on chromosome 7**E** [9]; *Psathyrostachys huashanica* chromosome 3**Ns** with the gene(s) for resistance to stripe rust [10]. Distant hybridization, including interspecific and intergeneric, was the first step to introduce elite alien genes to wheat. Many materials produced from distant hybridization, including amphidiploids and addition, substitution, as well as translocation lines with desirable exogenous genes, were considered as immediate materials for transferring alien chromosomal segments into wheat [11].

As one of the important wild relative genera of wheat, *Agropyron* Gaertn. possesses a basic **P** genome [12] and contains a large number of useful agronomical traits for wheat improvement, such as resistance to powdery mildew, barley yellow dwarf virus, leaf rust, stripe rust and stem rust [12–18], tolerance to drought [15,19] and low temperature [20], as well as more fertile tiller numbers per plant, spikelets and florets than wheat [7,12]. Therefore, the **P** genome can be used as a donor to provide desirable genes for wheat genetic improvement. To introduce these favorable genes to wheat, many domestic and international researchers have crossed *Agropyron* species with wheat. Smith and White [21,22] first began distant hybridization between *Agropyron* and wheat in the 1940s. Chen et al. [23] successfully hybridized *Triticum aestivum* Chinese Spring and tetraploid *Agropyron* for the first time in 1989. Li et al. [24–27] synthesized a series of intergeneric hybrids through wide hybridization and embryo rescue, and then obtained an array of wheat-*A*. *cristatum* addition lines. The production of wheat-*A*. *cristatum* disomic addition lines will be helpful for not only understanding the genetic constitution and genetic effects of the **P**-genome chromosomes under the background of common wheat but also providing the possibility of introducing *A*. *cristatum* genes into wheat for genetic improvement.

The alien disomic addition lines introduce some unfavorable genes for agronomic and end-use quality traits because of the ‘linkage drag’ during the introduction of the desirable gene. At the same time, the low fertility and genetic instability of alien addition lines make them unlikely to be directly useful in crop production [28]. Translocation lines, particularly some with small alien segments, were usually considered to be more stable and desirable because they lacked any linkage drag. Therefore, development of wheat-*A*. *cristatum* translocations makes it possible to transfer agronomically useful genes to wheat. Wheat-*A*. *cristatum* 6**P** translocation lines have been already obtained via the wheat-*A*. *cristatum* disomic addition line as a bridge material to introduce large numbers of florets and kernels per spike into wheat [6,29,30]. The development of wheat-*A*. *cristatum* 1.4**P** translocation lines makes it possible to introduce drought and cold tolerance genes to wheat [31]. A compensating Robertsonian translocation has been developed between *A*. *cristatum* and wheat, and it could be a useful source of leaf rust resistance in wheat [16].

It was observed that *Triticum aestivum* cv. ‘Fukuhokomugi’-*A*. *cristatum* 2**P** alien disomic addition line II-9-3 showed high resistance to powdery mildew and leaf rust compared with ‘Fukuhokomugi’ in our laboratory (Submitted). Therefore, the production of wheat-*A*. *cristatum* 2**P** translocation lines may transfer genes conferring resistance to powdery mildew and leaf rust from chromosome 2**P** into wheat.

The aim of this study was to produce various types of wheat*-A*. *cristatum* 2**P** translocation lines induced by ^60^Co-γ irradiation and gametocidal chromosomes 2**C**, and identify and characterize these translocation lines by GISH/FISH. These newly developed translocation lines will not only lay a solid groundwork for taking advantage of desirable genes on the chromosome 2**P** for wheat improvement, but also provide novel germplasms and valuable materials for studying gene expression, balance and interaction between different 2**P** chromosome segments of *A*. *cristatum* and common wheat.

## Materials and Methods

### Plant materials

Wheat-*A*. *cristatum* 2**P** disomic addition line II-9-3 (2*n* = 44) was obtained by hybridization between *A*. *cristatum* accession Z559 (2*n* = 4*x* = 28, **PPPP**, from Xinjiang, China) and *Triticum aestivum* cv. ‘Fukuhokomugi’ (2*n* = 6*x* = 42, **AABBDD**) and was inherited stably through several generations of backcrosses. This line was obtained and provided by Dr. Lihui Li of Chinese Academy of Agricultural Sciences. *T*. *aestivum* cv. ‘Chinese Spring’-*Aegilops cylindrica* Host 2**C** addition lines (CS-G2C) (2*n* = 44) were provided by Professor Jilin Li of Harbin Normal University, China.

### Induction techniques and cross combination

Wheat-*A*. *cristatum* 2**P** disomic addition line II-9-3 was overwintered in the field and transplanted into pots before jointing. The plants at the booting stage were irradiated with ^60^Co gamma rays at a dose of 20 Gray (Gy) and a dose rate of 0.5 Gy/min at the cobalt source chamber of Beijing University [30]. Fresh pollen collected from the donor parent ‘Fukuhokomugi’ was used to pollinate the irradiated spikes of addition line II-9-3, which had been artificially emasculated 1–3 days prior. Mature hybrid seeds were harvested and used to produce a M_1_BC_1_ population. Pollen collected from ‘Fukuhokomugi’ was used to pollinate the untreated spikes of wheat-*A*. *cristatum* 2**P** disomic addition line II-9-3 as a control.

Crosses were carried out using the CS-G2C addition line as the female parent and the wheat-*A*. *cristatum* 2**P** disomic addition line II-9-3 as the male parent. Mature hybrid seeds were harvested and selfed to produce a F_2_ population.

All the materials surveyed in this study were planted at the Chinese Academy of Agricultural Science Experiment Station in Beijing (39°57'13"N, 116°19'20"E) during the 2013–2014 growing season.

### Chromosome preparation

The exposed seeds were dipped into water in Petri dishes with double moistened filter papers at room temperature for one day, and then the water was absorbed by dry filter papers. The seeds were transferred into a refrigerator at 4°C for 48 h and germinated at 23°C in an incubator. Roots were sampled when the length of the roots was approximately 1.5–2.0 cm; these samples were pretreated in ice water for 24–48 h, fixed in a solution of absolute ethanol-acetic acid (3:1, v:v) at 4°C for 48 h, and then kept in 70% ethanol solution at -20°C. The chromosome slides were treated with 45% glacial acetic acid [32]. Cytological observations were made under a BX51 Olympus phase contrast microscope (Olympus Corp., Tokyo, Japan), and the images were captured using a digital camera. The slides were fixed using ultraviolet light in a TL-2000 Ultraviolet Translinker (Japan) for one minute when needed for GISH detection.

### GISH analysis

GISH was used to analyse the mitotic metaphase cells of the materials used in this study. *A*. *cristatum* and ‘Fukuhokomugi’ genomic DNA was isolated by a modified CTAB (hexadecyl trimethyl ammonium bromide) method [33]. The purity and concentration of DNA were measured using a spectrophotometer. The **P**-genomic DNA and ‘Fukuhokomugi’ genomic DNA were used as probe and block, at a ratio of 1:40, respectively, to identify the *A*. *cristatum* chromosomal fragments. The total genomic DNA of *A*. *cristatum* was labelled with digoxigenin-11-dUTP and used as a probe for GISH. The GISH procedure followed that described by Liu et al. [31]. The GISH images were observed under a Nikon Eclipse E600 (Japan) fluorescence microscope and captured with a CCD camera (Diagnostic Instruments, Inc., Sterling Heights, MI, USA).

### FISH analysis

FISH was used to characterize the translocated wheat chromosome, and dual-color FISH/GISH was employed by using repetitive DNA clones, i.e., pAs1, pHvG39, pSc119.2 and the total genomic DNA of *A*. *cristatum* as probes. The clones pAs1, pHvG39 and pSc119.2 were labelled with digoxigenin-11-dUTP, biotin-16-dUTP and biotin-16-dUTP, respectively, and were used as probes for dual-color FISH. Chromosomes were counterstained with DAPI (4’, 6-diamidino-2-phenylindole). The hybridization signals were examined using a Nikon Eclipse E600 (Japan) fluorescence microscope, and the FISH images were captured using a CCD camera. After FISH, the hybridization signals were washed with PBS (phosphate-buffered saline). The total genomic DNA of *A*. *cristatum* was labelled with digoxigenin-11-dUTP as a probe for subsequent GISH. Finally, according to the two hybridization results for the same cell, the homoeologous groups of the translocated wheat chromosomes were analysed by reference to the standard idiogram of the chromosomes of Chinese Spring wheat showing the locations of FISH signals [34,35].

## Results

### GISH detection of M_1_BC_1_ and F_2_ plants induced by ^60^Co-γ irradiation and gametocidal chromosome 2C

The GISH results were shown in Table 1. GISH analysis of 263 M_1_BC_1_ progenies from ^60^Co-γ irradiation showed that 39 plants had translocations between chromosome 2**P** and the wheat chromosomes, 204 plants had a whole chromosome 2**P** and 20 plants had no chromosome 2**P**. The translocation frequency induced by irradiation was 14.83%.

**Table 1:** GISH detection of the M_1_BC_1_ and F_2_ plants.

Type of plants	^60^Co-γ irradiation		Gametocidal chromosome 2C	
	No. of plants	Frequency of plants (%)	No. of plants	Frequency of plants (%)
**Alien translocation line**	39	14.83	10	6.76
**2P Disomic addition**	0	0	4	2.70
**2P Monosomic addition**	204	77.57	37	25.00
**No signal**	20	7.60	97	65.54
**Total**	263	100	148	100

Among the 148 F_2_ hybrid progenies of the CS-G2C and wheat-*A*. *cristatum* 2**P** addition lines, 51 plants had **P** chromosomal segments, accounting for 34.46% of the examined plants. Among them, there were 10 translocated plants (6.76%), 4 double **P** fragments addition plants (2.70%) and 37 single **P** fragment addition plants (25.00%). At the same time, neither translocations nor any other types of chromosomal structural changes were detected in any of the wheat-*A*. *cristatum* 2**P** disomic addition line/‘Fukuhokomugi’ F_1_ plants (control). This indicated that the translocation lines in this experiment totally resulted from the irradiation and gametocidal chromosome effect. Moreover, the frequency of alien translocation induced by irradiation was significantly higher than that induced by gametocidal chromosome 2**C**.

### The alien translocation types and frequencies of the M_1_BC_1_ and F_2_ progenies

Among the 411 M_1_BC_1_ and F_2_ progenies, 49 translocation plants were verified and various translocation types were obtained. The translocation types and frequencies between wheat and *A*. *cristatum* 2**P** chromosomes were shown in Table 2. The translocation plants were divided into two categories: the translocation plants with or without an intact chromosome 2**P**.

**Table 2:** The translocation types and frequencies of wheat-*A*. *cristatum* chromosome 2P translocation.

Type of 2P TL^a^		No. of TL	Frequency of TL (%)
Contain whole 2**P** chromosome	Large and small alien segmental	1	2.04
	Small alien segmental	2	4.08
Without whole 2**P** chromosome	Large alien segmental^b^ + Whole-arm	2	4.08
	Small alien segmental^c^ + Whole-arm^d^	2	4.08
	Two small alien segmental + Two intercalary	1	2.04
	Two small alien segmental+Intercalary^e^	1	2.04
	Whole arm + Intercalary	1	2.04
	Large and small alien segmental	10	20.41
	Whole-arm reciprocal	2	4.08
	Large alien segmental	10	20.41
	Small alien segmental	5	10.20
	Whole-arm	7	14.29
	Intercalary	3	6.12
	Chimera	2	4.08
Total		49	100

^a^ translocation line.

^b^ large alien segmental translocation indicates that the chromosome 2**P** segment is longer than one arm, the chromosome contains *A*. *cristatum* 2**P** centromere but no wheat centromere

^c^ small alien segmental translocation indicates that the chromosome 2**P** segment is shorter than one arm, the chromosome contains wheat centromere but no *A*. *cristatum* 2**P** centromere

^d^ whole-arm translocation indicates that both of the arms of the translocated chromosome are from wheat and *A*. *cristatum* respectively

^e^ intercalary translocation indicates that chromosome 2**P** segment is inserted into wheat chromosome arms.

**Fig 1:**
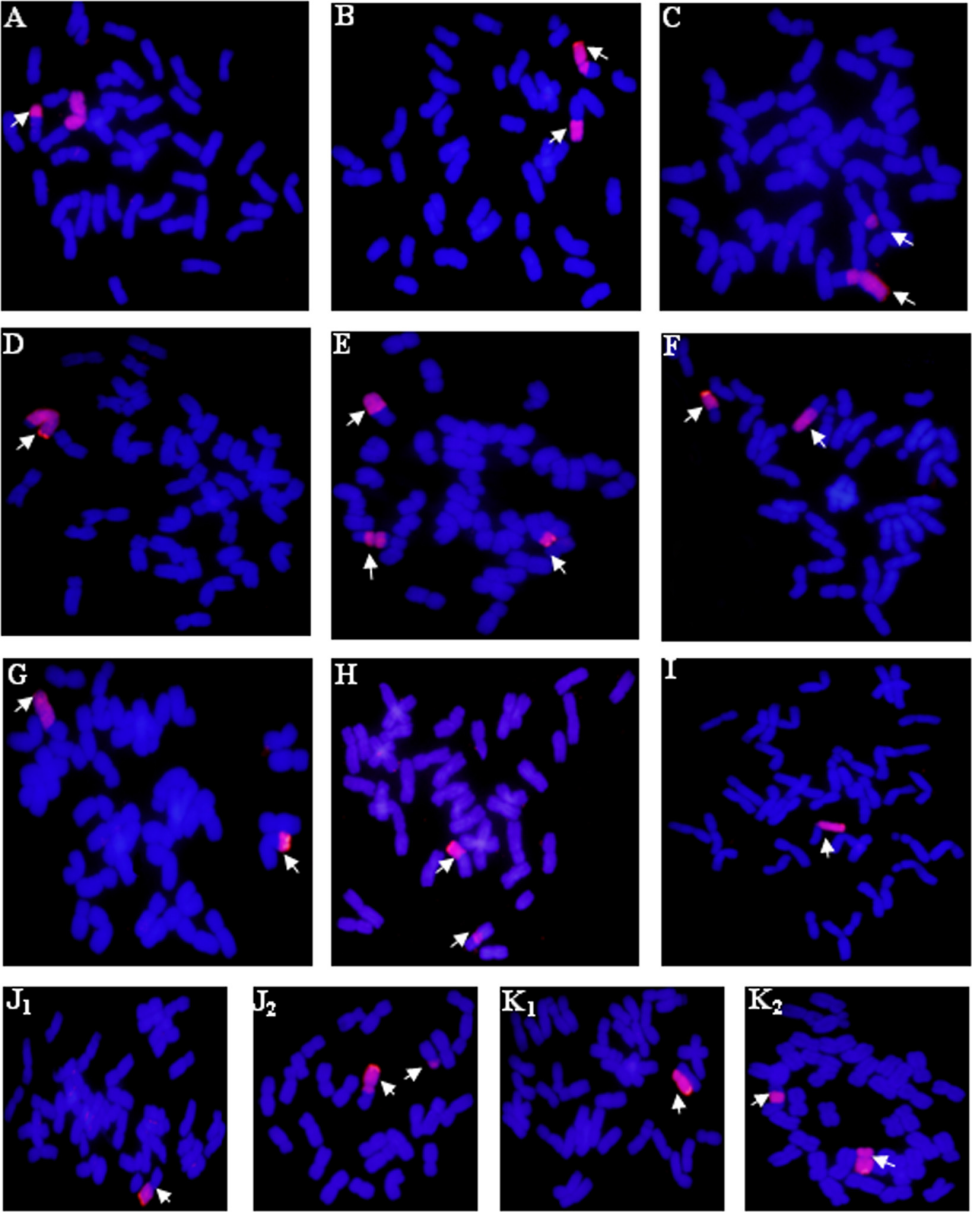
GISH patterns of some plants with wheat-*A*. *cristatum* 2P translocated chromosomes. *A*. *cristatum* genomic DNA was labelled with digoxigenin-11-dUTP red fluorescence and the wheat DNA was counterstained with DAPI blue fluorescence. Arrows show the translocated chromosomes. **A**: Small alien segmental translocation and a 2**P** chromosome. **B**: Large alien segmental and whole-arm translocation. **C**: Large and small alien segmental translocation. **D**: Large alien segmental translocation. **E**: Two small alien segmental and intercalary translocation. **F**: Whole-arm reciprocal translocation. **G**: Whole-arm and small alien segmental translocation. **H**: Whole arm and intercalary translocation. **I**: Whole-arm translocation. **J**_**1**_: Whole-arm translocation. **J**_**2**_: Large and small alien segmental translocation. **K**_**1**_: Deletion. **K**_**2**_: Intercalary translocation and deletion. **TL**: Translocation line.

There were 3 alien translocation plants with one intact 2**P**, including one plant (2.04%) with both small and large alien segmental translocation, as well as 2 plants (4.08%) with small alien segmental translocation (Fig 1A). There were 46 alien translocation plants without one intact 2**P**: 2 plants (4.08%) having large alien segmental and whole-arm translocation (Fig 1B), 10 plants (20.41%) having both large and small alien segmental reciprocal translocation (Fig 1C), 10 plants (20.41%) having large alien segmental translocation (Fig 1D), 1 plant (2.04%) with two small alien segmental and two intercalary translocations, 1 plant (2.04%) with two small alien segmental and one intercalary translocation (Fig 1E), 2 plants (4.08%) with two whole-arm reciprocal translocation (Fig 1F), 2 plants (4.08%) having small alien segmental and whole-arm translocation (Fig 1G), 1 plant (2.04%) with whole-arm and intercalary translocation (Fig 1H), 5 plants (10.20%) with small alien segmental translocation, 7 plants (14.29%) with whole-arm translocation (Fig 1I) and 3 plants (6.12%) with intercalary translocation. Moreover, we found 2 chimeras in plants 2**P**-189 and 2**P**-192. Plant 2**P**-189 had a whole-arm (Fig 1J_1_) or large and small alien segmental reciprocal translocation (Fig 1J_2_) in different root tip cells. Plant 2**P**-192 had a deletion (Fig 1K_1_) or intercalary translocation and deletion (Fig 1K_2_) in different root tip cells.

**Fig 2:**
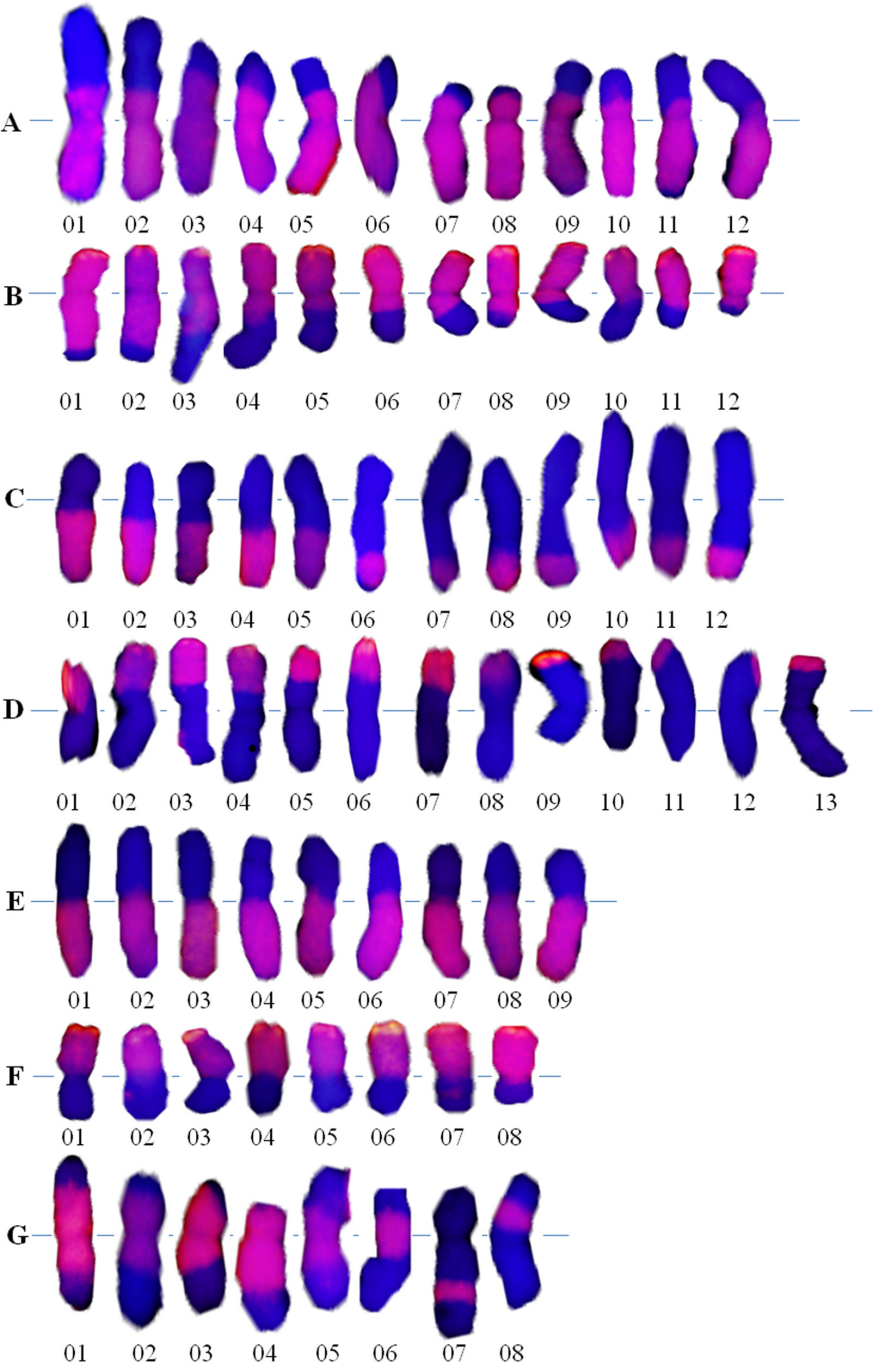
Grouping of the 74 wheat-*A*. *cristatum* 2P translocated chromosomes detected in the M_1_BC_1,_ F_2_ plants. The **P**-genomic DNA signal was red (wheat DNA was stained blue by DAPI). **A:** Large alien segmental translocation containing the whole 2**P** chromosome long arm. **B:** Large alien segmental translocation containing the whole 2**P** chromosome short arm. **C:** Small alien segmental translocation containing the partial 2**P** chromosome long arm. **D:** Small alien segmental translocation containing the partial 2**P** chromosome short arm. **E:** Whole arm translocation containing the whole 2**P** chromosome long arm. **F:** Whole arm translocation containing the whole 2**P** chromosome short arm. **G:** Intercalary translocation.

In this paper, we reported 74 translocated chromosomes including 24 large alien segmental translocations (LASTs), 25 small alien segmental translocations (SASTs), 8 intercalary translocations (ITs) and 17 whole arm translocations (WATs) (Fig 2). This result showed an ascending order of IT < WAT < LAST ≈ SAST in the occurrence frequency (Table 3). The ratio of SASTs (33.78%), which was the highest, was similar to that of LASTs (32.43%), indicating that SASTs and LASTs were easier to obtain. The ratio of ITs accounted for only 10.81% of the total translocations, indicating that intercalary translocations were the most difficult types to induce. At the same time, 74 translocated chromosomes differed in length. It showed that ^60^Co-γ irradiation and gametocidal chromosome 2C can randomly cause chromosome breakages and the breakpoints also tended to distribute randomly.

**Table 3:** Effect of ^60^Co-γ irradiation and gametocidal chromosome 2C on translocation breakpoints and types.

**TC**^**a**^ **type**	**LAST**^**b**^	**SAST**^**c**^	**IT**^**d**^	**WAT**^**e**^	**Total**
**No. of TC**	24	25	8	17	74
**Frequency of TC (%)**	32.43	33.78	10.81	22.97	100
**BFE**^f^	**IBF**^g^	**IBF**	**IBF**	**CBF**^h^	**Total**
**No. of BFE**	24	25	16	17	82
**Frequency of BFE (%)**	29.27	30.49	19.51	20.73	100

^a^ translocation chromosome

^b^ large alien segmental translocation

^c^ small alien segmental translocation

^d^ intercalary translocation

^e^ whole arm translocation

^f^ breakage-fusion event

^g^ interstitial breakage-fusion

^h^ centric breakage-fusion.

The 74 translocated chromosomes involved 82 breakage-fusion events, including 65 in interstitial regions (79.27%) and 17 in centric regions (20.73%) (Table 3; Fig 2), indicating that ^60^Co-γ irradiation and gametocidal chromosome 2C had very high efficiency in inducing interstitial breakage. Sixty-six translocations (89.8%, including 17 WATs, 24 LASTs and 25 SASTs) involved one-breakage fusion events, while only 8 ITs involved two-breakage fusion events. This indicated that the major effect of irradiation and gametocidal chromosome 2**C** was to induce one-breakage fusion events.

### Identification of the wheat translocated chromosomes with *A*. *cristatum* chromosome 2P

To identify the wheat translocated chromosomes with *A*. *cristatum* chromosome 2**P**, dual-color FISH/GISH was performed in wheat root-tip cells. The FISH results were shown in Table 4. Twenty-three types of translocated chromosomes were identified in the eighteen wheat-*A*. *cristatum* alien chromosomal translocation lines. *A*. *cristatum* 2**P** chromosome fragments were translocated with wheat chromosomes 1**A**, 2**A**, 3**A**, 4**A**, 5**A**, 6**A**, 7**A**, 3**B**, 5**B**, 7**B**, 1**D**, 4**D** and 6**D**.

**Table 4:** Identification of the translocated wheat chromosomes.

TL	Type of TL	pSc119.2	pHvG39	pAs1	Wheat chromosome
2**P**-23	Large and small alien segmental	/^a^	+^b^	-^c^	3**A**, 5**B**
2**P**-35	intercalary	/	-	-	7**A**
2**P**-40	Small alien segmental	/	-	+	4**D**
2**P**-43	Whole-arm	/	-	-	1**A**
2**P**-48	Large and small alien segmental	/	-	+	1**D**,7**A**
2**P**-55	Whole-arm reciprocal	/	-	-	5**A**
2**P**-80	Whole-arm	/	-	-	3**A**
2**P**-116	Small alien segmental	/	+	-	7**B**
2**P**-122	Small alien segmental	/	+	-	4**A**
2**P**-167	Small alien segmental	/	-	-	6**A**
2**P**-173	Small alien segmental	/	-	+	6**D**
2**P**-187	Large alien segmental	/	-	-	3**A**
2**P**-190	Whole-arm + Large alien segmental	/	+	-	2**A**, 7**A**
2**P**-205	Whole-arm	/	-	+	4**D**
2**P**-213	Whole-arm	+	/	-	3**B**
2**P**-269	Large alien segmental	/	+	-	7**A**
2**P**-355	Large alien segmental	/	-	+	4**D**
2**P**-367	Large and small alien segmental	/	-	-	5**A**

^a^ none detected

^b^ positive

^c^ negative.

**Fig 3:**
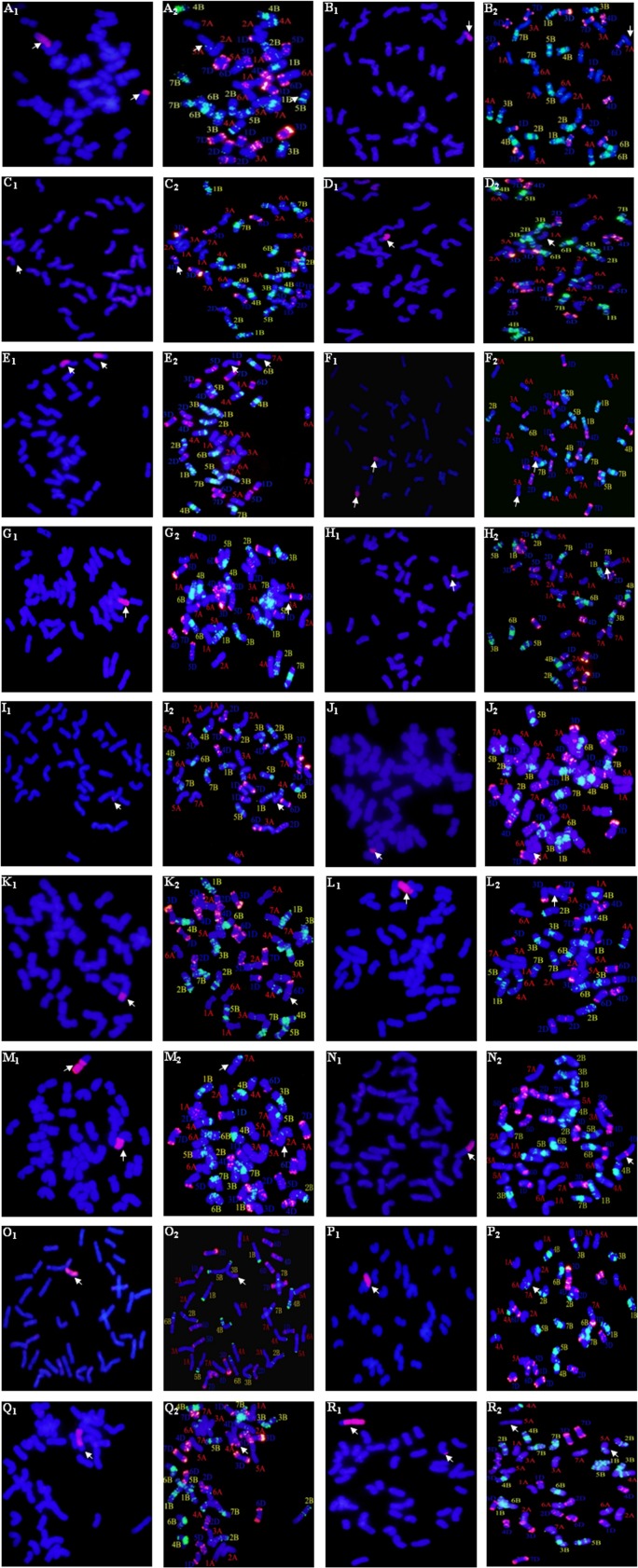
Dual-color FISH/GISH identification of the homoeologous group of partially translocated wheat chromosomes. **A**_**1**_**-R**_**1**_: GISH patterns of wheat-*A*. *cristatum* translocation lines. Total genomic DNA of *A*. *cristatum* was labelled with digoxigenin-11-dUTP and visualized with red fluorescence. Chromosomes were counterstained with DAPI and visualized with blue fluorescence. **A**_**2**_**-R**_**2**_: FISH patterns of wheat-*A*. *cristatum* translocation lines. **A**_**2**_**-N**_**2**_, **P**_**2**_**-R**_**2**_: The repetitive sequence clone pHvG39 was labelled with biotin-16-dUTP and visualized with green fluorescence; **O**_**2**_: The repetitive sequence clone pSc119.2 was labelled with biotin-16-dUTP and visualized with green fluorescence. The repetitive sequence clone pAs1 was labelled with digoxigenin-11-dUTP and visualized with red fluorescence. Chromosomes were counterstained with DAPI and visualized with blue fluorescence. Arrows point to the translocated chromosomes. **A.** 2**P**-23; **B.** 2**P**-35; **C.** 2**P**-40; **D.** 2**P**-43; **E.** 2**P**-48; **F.** 2**P**-55; **G.** 2**P**-80; **H.** 2**P**-116; **I.** 2**P**-122; **J.** 2**P**-167; **K.** 2**P**-173; **L.** 2**P**-187; **M.** 2**P**-190; **N.** 2**P**-205; **O.** 2**P**-213; **P.** 2**P**-269; **Q.** 2**P**-355; **R.** 2**P**-367.

We performed dual-color FISH/GISH using **P** genomic DNA and the clone pAs1 labelled with digoxigenin-11-dUTP, the clones pHvG39 and pSc119.2 labelled with biotin-16-dUTP. Plant 2**P**-23 has 2 translocated chromosomes; the small alien segmental translocation of 2**P**-23 showed an obvious green fluorescent signal near the centromeric region of the short arm and a faint green signal in the middle of the long arm; the arm ratio is large. Accordingly, it was a 5**B**S.L-2**P** translocation with a small 2**P** fragment. Further, the large alien segmental translocation of 2**P**-23 exhibited a faint green signal at the end of the arm; it was a 3**A**-2**P** translocation with a large 2**P** fragment (Fig 3A_1_ and 3A_2_). Plant 2**P**-35 contained a 7**A-**2**P**L.2**P**S-7**A** intercalary translocation (Fig 3B_1_ and 3B_2_). Plant 2**P**-40 contained a 4**D**L.4**D**S-2**P**L small-segment translocation (Fig 3C_1_ and 3C_2_). Plant 2**P**-43 contained a 1**A**-2**P**S whole-arm translocation (Fig 3D_1_ and 3D_2_). Plant 2**P**-48 was a dual heterozygous translocation plant containing 1**D**-2**P**L and 7**A**-2**P**S (Fig 3E_1_ and 3E_2_). Plant 2**P**-55 had a 5**A**-2**P** reciprocal translocation with 2**P** fragments (Fig 3F_1_ and 3F_2_). Plant 2**P**-80 contained a 3**A**-2**P**L whole-arm translocation (Fig 3G_1_ and 3G_2_). Plant 2**P**-116 contained a 7**B**-2**P** small-segment translocation (Fig 3H_1_ and 3H_2_). Plant 2**P**-122 contained a 4**A**L.4**A**S-2**P**L small-segment translocation (Fig 3I_1_ and 3I_2_). Plant 2**P**-167 contained a 6**A**S.6**A**L-2**P**L small-segment translocation (Fig 3J_1_ and 3J_2_). Plant 2**P**-173 contained a 6**D**S.6**D**L-2**P**L small-segment translocation (Fig 3K_1_ and 3K_2_). Plant 2**P**-187 was a 3**A**-2**P**S.2**P**L large-segment translocation with a large 2**P** segment (Fig 3L_1_ and 3L_2_). Plant 2**P**-190 was a dual heterozygous translocation plant containing 2**A**-2**P**L and 7**A**-2**P**S (Fig 3M_1_ and 3M_2_). Plant 2**P**-205 contained a 4**D**-2**P**L whole-arm translocation (Fig 3N_1_ and 3N_2_). Plant 2**P**-213 contained a 3**B**-2**P**L whole-arm translocation (Fig 3O_1_ and 3O_2_). Plant 2**P**-269 was a 7**A**-2**P**S.2**P**L large-segment translocation with a large 2**P** segment (Fig 3P_1_ and 3P_2_). Plant 2**P**-355 was a 4**D**-2**P**S.2**P**L large-segment translocation with a large 2**P** segment (Fig 3Q_1_ and 3Q_2_). Plant 2**P**-367 had a 5**A**-2**P** reciprocal translocation with 2**P** fragments (Fig 3R_1_ and 3R_2_).

## Discussion

Chromosome translocations can be induced by *Ph*-system, tissue culture, gametocidal chromosomes and ionization irradiation. Allosyndetic associations between wheat and *A*. *cristatum* are rare [23,36–38]; consequently, homoeologous pairing between wheat and *A*. *cristatum* chromosomes rarely takes place even when induced with a *Ph*-system [39]. Translocations induced by tissue culture are very technical and troublesome because they require a long period to obtain regenerative plants [40]. Compared with the first two approaches, the gametocidal chromosome from *Aegilops cylindrica* Host was successfully used to induce chromosome mutations in common wheat in nature. These chromosome variations could be transmitted to stable offspring [41], which was considered as an effective method to induce chromosomal structural variations [42]. Ionizing irradiation can induce chromosome breakage randomly and yield many translocation types including small segment translocations and intercalary translocations [43], which has been widely used in transferring useful genes from wild relatives to wheat for the improvement of resistance or tolerance to biotic and abiotic stresses [8,29,44,45]. In our study, forty-nine 2**P** translocated plants were successfully obtained, which was the first study to report such a finding. Abundant types of alien chromosome translocations were induced by both ionizing irradiation and gametocidal chromosome 2**C**, which indicated that these two methods were useful for producing wheat-*A*. *cristatum* 2**P** translocations.

Ionizing irradiation has been proven as an effective method to induce chromosomal translocations [46]. Dry seed, plants at meiosis and spikes at the pollen stage can be used as irradiated materials [29,30,47]. The present study showed that irradiation of plants at meiosis was highly effective. We only pollinated irradiated spikes using fresh pollen from untreated plants. More M_1_BC_1_ seeds can be obtained as long as donor parents (untreated plants) have sufficient pollen. Furthermore, the treatment of booting stage plants in the pots was more convenient than that on spikes. Additionally, chromosomal structural variants can be directly identified in the M_1_BC_1_ generation by GISH.

To date, many translocation lines have been screened and identified involving three wheat-*A*. *cristatum* disomic addition lines [29–31]. A new strategy to rapidly produce a large number of translocations between wheat and *A*. *cristatum* has been developed [30]. This strategy was used to induce additional four wheat-*A*. *cristatum* disomic addition lines and to produce wheat-*A*. *cristatum* alien translocation lines with different breakpoints and **P** segments of different sizes. The translocation lines can be further used to construct a deletion bin map of each **P** chromosome.

The transfer of desirable genes from the tertiary gene pool could be an efficient way to increase genetic diversity and improve cultivated wheat [28,48]. Translocation lines, especially small alien segment translocations, would be genetically more stable and desirable [49]. The production of small alien segment translocations makes it possible to transfer useful genes from wild relatives to wheat compared with the low fertility and genetic instability of addition and substitution lines [50–52]. *A*. *cristatum* has many potentially valuable traits that can be used in wheat improvement, so it is important to produce wheat-*A*. *cristatum* 2**P** small-segment translocations to transfer useful genes to wheat for broadening wheat genetic diversity. Huang et al. [53] identified two homozygous wheat-*A*. *cristatum* 6**P** small intercalary translocation lines. These lines did not carry undesirable genes and had a good compensation effect of *A*. *cristatum* chromatin, which further confirmed that the wheat-*A*. *cristatum* small alien segments translocations were desirable. Twenty-five small fragment alien translocations were obtained in the present study by ionizing irradiation and gametocidal chromosome 2**C**, which would make it possible to utilize desirable genes from *A*. *cristatum* 2**P** chromosome.

Many wheat-alien chromosomal translocation lines carrying desirable genes have been reported in recent years [8,54,55], but some have not been fully utilized in wheat breeding due to the incomplete compensation for the replaced wheat chromosomal segments. Only well-compensating translocations were beneficial in wheat improvement. Until now, only a few exogenous desirable genes have played an important role in wheat breeding [4,56]. It is of great significance to explore the evolutionary relatedness, homoeologous relationships and degree of colinearity between wheat and alien chromosomes [4,52,57]. *Agropyron* are cross-pollinating plants. The tetraploid *A*. *cristatum* derived from the hybridizations between diploid *A*. *cristatum* and *A*. *mongolicum*. Although the diploid *A*. *cristatum* and *A*. *mongolicum* contained the same basic **P** genome, their **P** genomes exhibited rearrangements and variation [58,59]. Han et al. [17] detected the genetic rearrangement of **P** genomes by identifying four different types of wheat-*A*. *cristatum* 6**P** disomic addition lines and speculated that genomic rearrangements may occur in the wheat-*A*. *cristatum* addition line. Accordingly, we speculated that *A*. *cristatum* chromosome 2**P** may have genetic arrangement to some extent. Although the *A*. *cristatum* chromosome 2**P** fragment was translocated to wheat homoeologous groups (1, 3, 4, 5, 6 and 7) in this study, some wheat-*A*. *cristatum* 2**P** translocation lines were not agronomically poor in the field environment. Thus, we could not exclude the possibility that the wheat-*A*. *cristatum* 2**P** translocation lines may have a subordinate complementary relationship with wheat homoeologous groups (1, 3, 4, 5, 6 and 7) and that the offspring may have a partial compensation effect because of genomic rearrangement. The present study showed that *A*. *cristatum* chromosome 2**P** was translocated to all three wheat genomes and that the wheat **A** genome had the highest recombination frequency. The results were inconsistent with previous studies, which showed that the wheat **B** genome possessed the highest number of chromosome arrangements [60]. The translocation lines obtained in this study differed in breakpoint locations and alien segment lengths. The same 2**P** chromosome segment could be transferred to different wheat chromosomes/genomes. Different 2**P** chromosome segments could also be transferred to the same wheat chromosome/homoeologous group. These lines would be useful in studies to better understand recombination, interaction and genetic balance between wheat chromosomes and the *A*. *cristatum* 2**P** chromosome. In addition, they could provide a theoretical basis for the utilization of desirable genes of the chromosome 2**P** in wheat breeding.

Powdery mildew and leaf rust, caused by *Blumeria graminis* f. sp. *tritici* (Bgt) and *Puccinia recondita* f. sp. *tritici*, respectively, are two devastating diseases that cause severe yield losses in most of the wheat production areas [61,62]. Powdery mildew can cause yield losses up to 50% [63], whereas leaf rust caused yield losses ranging from 40% [64] to 70% [65]. Breeding resistant varieties is the most economical and effective way to control these diseases. To date, approximately 42 loci with more than 70 alleles conferring resistance to powdery mildew genes [66] and more than 70 loci for leaf rust resistance genes [67] have been reported. Unfortunately, many of these resistance genes usually become ineffective when new pathogen variants emerge due to co-evolution of the host and pathogen [68]. Hence, there is an urgent need to identify new and effective sources of resistance for wheat improvement. It has been observed that the wheat-*A*. *cristatum* 2**P** addition line possessed high resistance to powdery mildew and leaf rust compared with ‘Fukuhokomugi’ in our laboratory (Submitted). By preliminary phenotypic evaluation, newly developed 2**P** alien translocation lines 2**P**-173 and 2**P**-205 were highly resistant to powdery mildew (S1 Fig) and the translocation line 2**P**-205 was highly resistant to leaf rust (S2 Fig). This showed that the *A*. *cristatum* 2**P** chromosome harbored genes conferring high resistance to powdery mildew and leaf rust. Furthermore, segregating populations involved in wheat-*A*. *cristatum* 2**P** alien translocation lines with different breakpoints are being developed. These will lay the foundation for locating and cloning novel powdery mildew and leaf rust resistance genes and providing new germplasm accessions for wheat genetic improvement.

In summary, the wheat-*A*. *cristatum* 2**P** alien chromosomal translocations were successfully induced and numerous translocation lines that possessed whole-arm, segmental and intercalary translocations were obtained for the first time in this study. These newly developed translocation lines will not only lay the foundation for mapping and cloning of powdery mildew and leaf rust resistant genes on the *A*. *cristatum* 2**P** chromosome, but also provide excellent germplasm resources and ideal experimental materials for breeding application and basic research.

## Supporting Information

S1 Fig Preliminary evaluation of powdery mildew resistance of the newly developed wheat-*A*. *cristatum* translocation lines and their parents. 1: Wheat-*A*. *cristatum* alien 2**P** disomic addition line II-9-3. 2: Wheat-*A*. *cristatum* 2**P** alien translocation line 2**P**-205. 3: Wheat-*A*. *cristatum* 2**P** alien translocation line 2**P**-173. 4: Fukuhokomugi. 5: Zhongzuo9504.
(TIF)

S2 Fig Preliminary evaluation of leaf rust resistance of the newly developed wheat-*A*. *cristatum* translocation line and their parents. 1: Wheat-*A*. *cristatum* alien 2**P** disomic addition line II-9-3. 2: Wheat-*A*. *cristatum* 2**P** alien translocation line 2**P**-205. 3: Fukuhokomugi.
(TIF)
