# Supplementary figures and images for: Production and Identification of Wheat-Agropyron cristatum 2P Translocation Lines
Source: PLoS One. 2016 Jan 5;11(1):e0145928. doi: 10.1371/journal.pone.0145928 (PMC4701160; doi:10.1371/journal.pone.0145928)

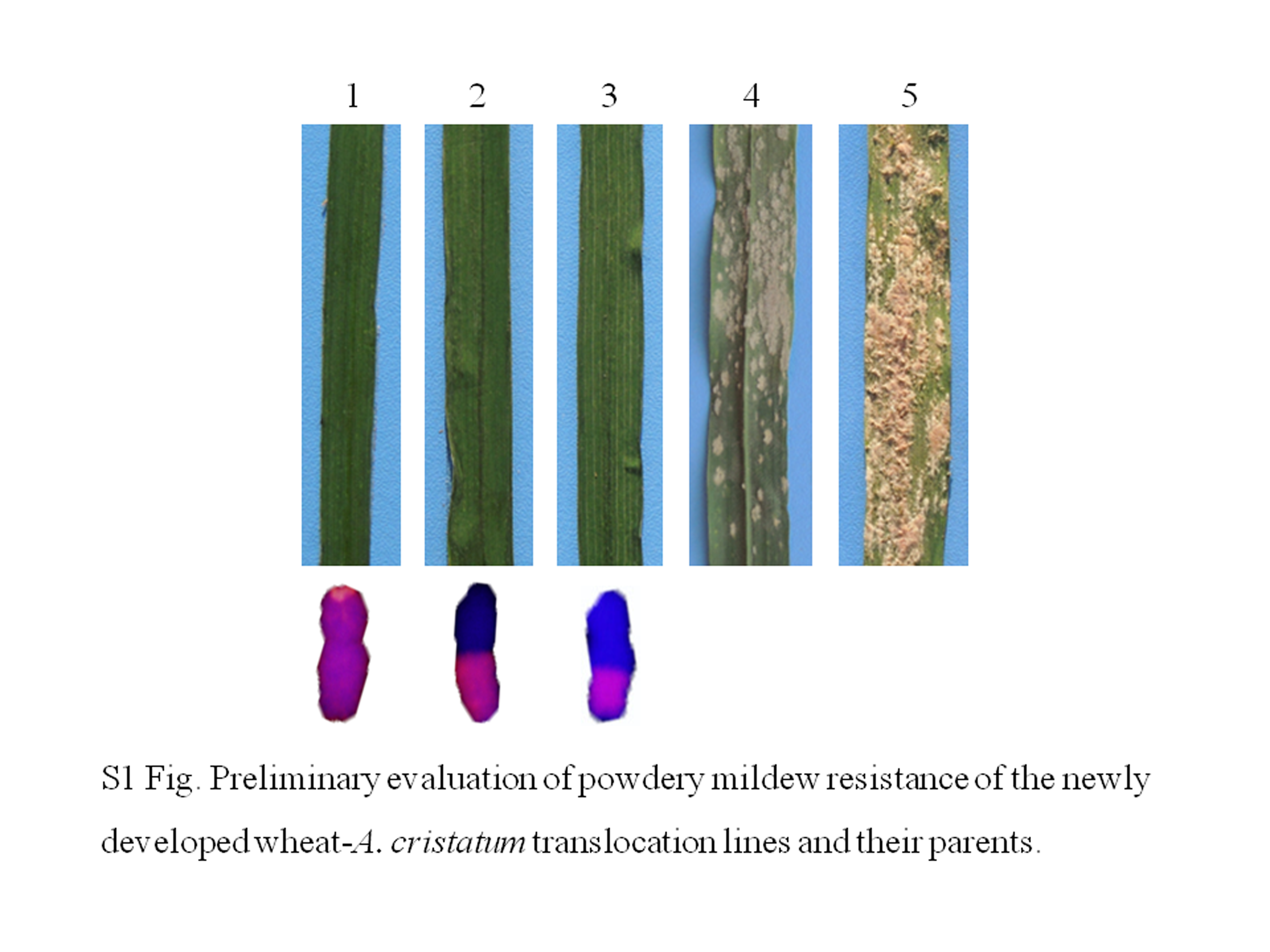

Supplement: S1 Fig — 1: Wheat-A. cristatum alien 2P disomic addition line II-9-3. 2: Wheat-A. cristatum 2P alien translocation line 2P-205. 3: Wheat-A. cristatum 2P alien translocation line 2P-173. 4: Fukuhokomugi. 5: Zhongzuo9504. (TIF) [file pone.0145928.s001.tif]

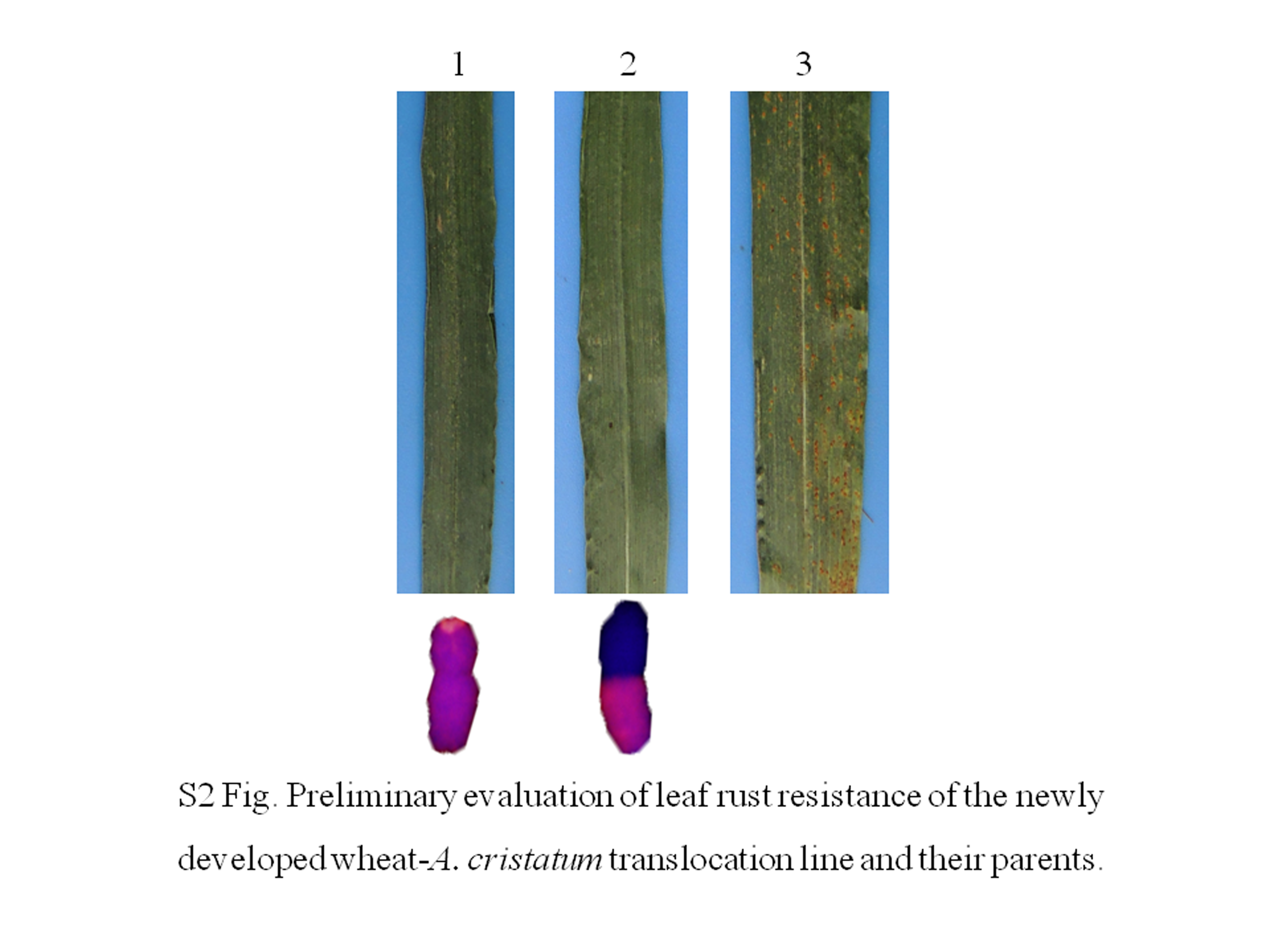

Supplement: S2 Fig — 1: Wheat-A. cristatum alien 2P disomic addition line II-9-3. 2: Wheat-A. cristatum 2P alien translocation line 2P-205. 3: Fukuhokomugi. (TIF) [file pone.0145928.s002.tif]
